# Canakinumab in the treatment of systemic juvenile idiopathic arthritis: a retrospective single center study in China

**DOI:** 10.3389/fped.2024.1349907

**Published:** 2024-03-14

**Authors:** Xiaona Zhu, Ruohang Weng, Yanyan Huang, Yongbin Xu, Jun Yang, Tingyan He

**Affiliations:** Department of Rheumatology and Immunology, Shenzhen Children’s Hospital, Shenzhen, China

**Keywords:** canakinumab, interleukin-1, systemic juvenile idiopathic arthritis, macrophage activation syndrome, cytokine

## Abstract

**Objective:**

Systemic juvenile idiopathic arthritis (sJIA) is characterized by excessive production of proinflammatory cytokines. As an anti-IL-1 agent, canakinumab has been approved in the USA and Europe for the treatment of sJIA patients aged ≥2 years. However, the use of canakinumab has never been reported in China. In this study, we aimed to assess the efficacy and safety of canakinumab in Chinese patients with sJIA.

**Methods:**

A total of 11 patients with sJIA who were treated with canakinumab were included in this study. Clinical data were collected retrospectively from medical records. Efficacy was evaluated by the systemic juvenile arthritis disease activity score (sJADAS). The follow-up was performed at canakinumab initiation, at months 1, 3, 6, 9 and 12, or at the last follow-up.

**Results:**

Of the 11 patients enrolled, 91.0% (10/11) had previously received treatment with tocilizumab. The mean duration of canakinumab was 9 (3–18) months. 45.5% (5/11) of patients showed complete response, 45.5% (5/11) showed partial response, and 9.0% (1/11) showed no response. 18.2% (2/11) experienced disease flare during the treatment with canakinumab. 81.8% (9/11) of patients successfully reduced the dose of corticosteroids, with six discontinuing corticosteroids. 45.6% (5/11) of patients experienced infection. No serious adverse events occurred during the treatment with canakinumab.

**Conclusions:**

Canakinumab may be effective and tolerable for Chinese sJIA patients, helping to reduce the dosage of corticosteroids. However, additional researches on large samples are required to evaluate its efficacy and safety.

## Introduction

Systemic juvenile idiopathic arthritis (sJIA) is characterized by arthritis, spiking fever, evanescent erythematous rash, lymphadenopathy, hepatomegaly, splenomegaly and/or serositis, in combination with a substantial increase in the inflammatory parameters, such as erythrocyte sedimentation rate (ESR), C-reactive protein (CRP) level, ferritin level and so on. Macrophage activation syndrome (MAS) is the most severe and life-threatening complication of sJIA, which occurs in 10%–30% of sJIA patients (1).

The pathogenesis of sJIA is not well understood. Previous studies suggest that inflammation in sJIA is closely related to innate immune dysregulation and driven by the overproduction of proinflammatory cytokines, such as interleukin (IL)-1β, IL-6, and IL-18. MAS is caused by activated macrophages and cytotoxic T cells, leading to cytokine storm with increased levels of IL-18, interferon-*γ* and IL-6 (2). Conventional treatments for sJIA include corticosteroids and tocilizumab. Although many patients respond to tocilizumab, a subset of patients continue to suffer refractory sJIA or sJIA-MAS in our country. IL-1 family of cytokines plays an crucial role in the inflammatory process of sJIA. Anti-IL-1 therapy is effective in sJIA patients without MAS and those with MAS (3).

Canakinumab is a fully human anti-IL-1β monoclonal antibody that selectively binds to IL-1β and prevents IL-1β-induced inflammatory mediator production. Previous studies have demonstrated the efficacy and safety of canakinumab in sJIA, suggesting its role in the dosage reduction of corticosteroids (4, 5). However, the use of canakinumab has never been reported in China. In this study, we described the cohort of Chinese patients with sJIA receiving canakinumab to evaluate its efficacy and potential adverse effects.

## Materials and methods

### Patient cohort and study approval

A retrospective analysis was performed on 11 patients who suffered from sJIA receiving the treatment with canakinumab from February 2022 to August 2023 in Shenzhen Children's Hospital. The diagnosis of sJIA was based on the International League Against Rheumatism classification criteria (6). The diagnosis of sJIA-associated MAS was based on 2016 classification criteria (7). Patients with the monogenic autoinflammatory syndrome were excluded by whole-exon sequencing. Data of age, gender, treatments, disease course, adverse effects, clinical manifestations, and laboratory findings were extracted from the medical electronic database at canakinumab initiation, at 1, 3, 6, 9 and 12 months or at the last follow-up. Laboratory tests included blood count, liver and kidney function tests, CRP, ESR, ferritin and fibrinogen levels. The study was approved by the ethics committee of Shenzhen Children's Hospital. The legal guardians of all patients had signed the informed consent for the study and treatment with canakinumab.

### Disease activity score and response to canakinumab

The disease activity rating scale of sJIA patients was evaluated according to sJADAS (8, 9). It included physician global assessment of overall activity, patient/parent global assessment of well-being, count of active joints, ESR or CRP level, and the modified systemic manifestation score.

Complete response was defined as sJADAS < 3 points, complete resolution of clinical SJIA-related symptoms, and normalization of laboratory parameters, especially inflammatory markers. Partial response was defined as a decrease in sJADAS but still ≥ 3 points, the persistence of some SJIA-related manifestations, and/or elevation of inflammatory markers. No response was defined as no clinically relevant improvement within three months after the use of canakinumab. Disease flare was defined as an increase in sJADAS, more sJIA related symptoms, or abnormal laboratory values (8, 10).

### Statistical analysis

Statistical analysis was performed using GraphPad Prism software (version 8.0.1, GraphPad Inc.). Data were expressed as median (minimum-maximum range). Paired t-test were used to compare two groups. A value of *p *< 0.05 was considered statistically significant.

## Results

### Patient disposition and baseline characteristics

The study group included 11 children (7 males and 4 females). The median age at onset of sJIA was 7.9 (1–14) years. The median age at canakinumab initiation was 9.2 (1.5–14.4) years. At the start of canakinumab treatment, all patients presented with active disease [median sJADAS 22.2 (17.2, 30.5)]. Four patients developed MAS at disease onset (Table 1).

**Table 1:** Baseline demographic and disease characteristics.

Characteristics, median (min,max), unless specified	Canakinumab (*n* = 11)
Age (years)	9.75 (1.9,15.45)
Male, *n* (%)	7 (63.6)
sJIA onset (years)	7.9 (1,14)
Fever, *n* (%)	11 (100)
Arthritis, *n* (%)	10 (90.9)
Rash, *n* (%)	8 (72.7)
MAS, *n* (%)	4 (36.4)
Oral prednisone equivalent dose (mg/kg/day)	0.60 (0,2.67)
Oral prednisone equivalent dose, *n* (%)	
>0.4 mg/kg/day	8 (72.7)
>0 to ≤0.4 mg/kg/day	2 (18.2)
Treatments before canakinumab, *n* (%)	
MTX	6 (54.5)
JAKi	8 (72.7)
Tocilizumab	10 (91.0)
TNF inhibitor	3 (27.3)
Anakinra	1 (9.1)
Thalidomide	1 (9.1)
Concomitant use of canakinumab, *n* (%)	
MTX	4 (36.4)
JAKi	6 (54.5)
Thalidomide	1 (9.1)
sJADAS	22.2 (17.2,30.5)

MAS, macrophage activation syndrome; MTX, methotrexate; JAKi, janus kinase inhibitors; TNF, tumor necrosis factor; sJADAS, the systemic juvenile arthritis disease activity score.

Before canakinumab initiation, all patients received at least one immunosuppressant or biological DMARD (bDMDARD). The most commonly used medication was tocilizumab (91.0%, 10/11). Only Patient 6 received anakinra. Janus kinase inhibitors (JAKi) and methotrexate (MTX) were used in 72.7% (8/11) and 54.5% (6/11), respectively (Table 1).

At the initiation of canakinumab treatment, most patients (91%, 10/11) received oral corticosteroids. The median equivalent dose of prednisone was 0.60 (0–2.67) mg/kg/day. 72.7% (8/11) of patients received at least 0.4 mg/kg/day. JAKi was given to 45.5% (5/11) of patients. Concomitant use of MTX was in five patients. Patient 4 was treated concomitantly with thalidomide (Tables 1, 2).

**Table 2:** Treatment with canakinumab in SJIA patients.

Patients	Concomitant treatments at canakinumab initiation	canakinumab dosing	canakinumab duration (months)	canakinumab at last follow-up	Response at canakinumab duration	Treatments at last follow-up	Remaining symptoms at last follow-up	Remaining abnormal laboratory findings at last follow-up	Infectious adverse effects at canakinumab duration	Other adverse side effects at canakinumab duration
1	prednison (40 mg/d), ruxolitinib 5 mg bid	first dosage 8 mg/kg followed by 4 mg/kg q4w→ 4 mg/kg q8w	18	ongoing	Complete response	MTX	No	No	Upper respiratory tract infection, Acute cervical lymphadenitis	No
2	dexamethasone (7.5 mg/d), ruxolitinib 5 mg bid	4 mg/kg q4w→ 4 mg/kg q8w	14	ongoing	Complete response	No	No	No	Upper respiratory tract infection	No
3	prednison (10 mg/d)	6 mg/kg q4w→5 mg/kg q8w	15	ongoing	Complete response	No	No	No	Upper respiratory tract infection, influenza	No
4	dexamethasone (2.25 mg/d), thalidomide 25 mg bid	2.7 mg/kg q2w→2.5 mg/kg q5w	12	ongoing	Partial response	MTX	Occasional rash	No	No	No
5	prednison (15 mg/d)	5 mg/kg q4w→5 mg/kg q6w→5 mg/kg q4w→ discontinue	9	Discontinuation for disease flare and persistent joint swelling	Partial response	prednison (10 mg/d), MTX,	Occasional rash, flare	ESR↑, CRP↑	Upper respiratory tract infection, Acute gastroenteritis	No
6	prednison (10 mg/d), MTX 12.5 mg qw, tofacitinib 5 mg qd	3.5 mg/kg q4w→3.5 mg/kg q2w→discontinue	6	Discontinuation for recurrent disease flare and poor compliance	No response, recurrent disease flare	prednison (10 mg/d), cyclosporin 75 mg bid, tofacitinib 5 mg qd	Rash, recurrent flare	FER↑	No	No
7	prednison (40 mg/d), MTX 10 mg qw	6 mg/kg q4w→6 mg/kg q9w	9	ongoing	Complete response	MTX	No	No	No	No
8	prednison (30 mg/d), MTX 15 mg qw	2 mg/kg q2w→2 mg/kg q5w	9	ongoing	Complete response	MTX	No	No	No	No
9	prednison (2.5 mg/d), MTX 5 mg qw	4 mg/kg q4w	3	ongoing	Partial response	prednison (1.25 mg/d), ruxolitinib	Occasional rash	No	Upper respiratory tract infection	No
10	prednison (7.5 mg/d), ruxolitinib 15 mg/d	5 mg/kg q4w	3	ongoing	Partial response	prednison (5 mg/d), ruxolitinib	Occasional rash	No	No	No
11	MTX 10 mg qw, tofacitinib 5 mg qd	4 mg/kg q4w	3	ongoing	Partial response	MTX, tofacitinib	No	ESR↑	No	No

MTX, methotrexate; FER, ferritin; ESR, erythrocyte sedimentation rate.

### Efficacy of canakinumab in sJIA

**Figure 1:**
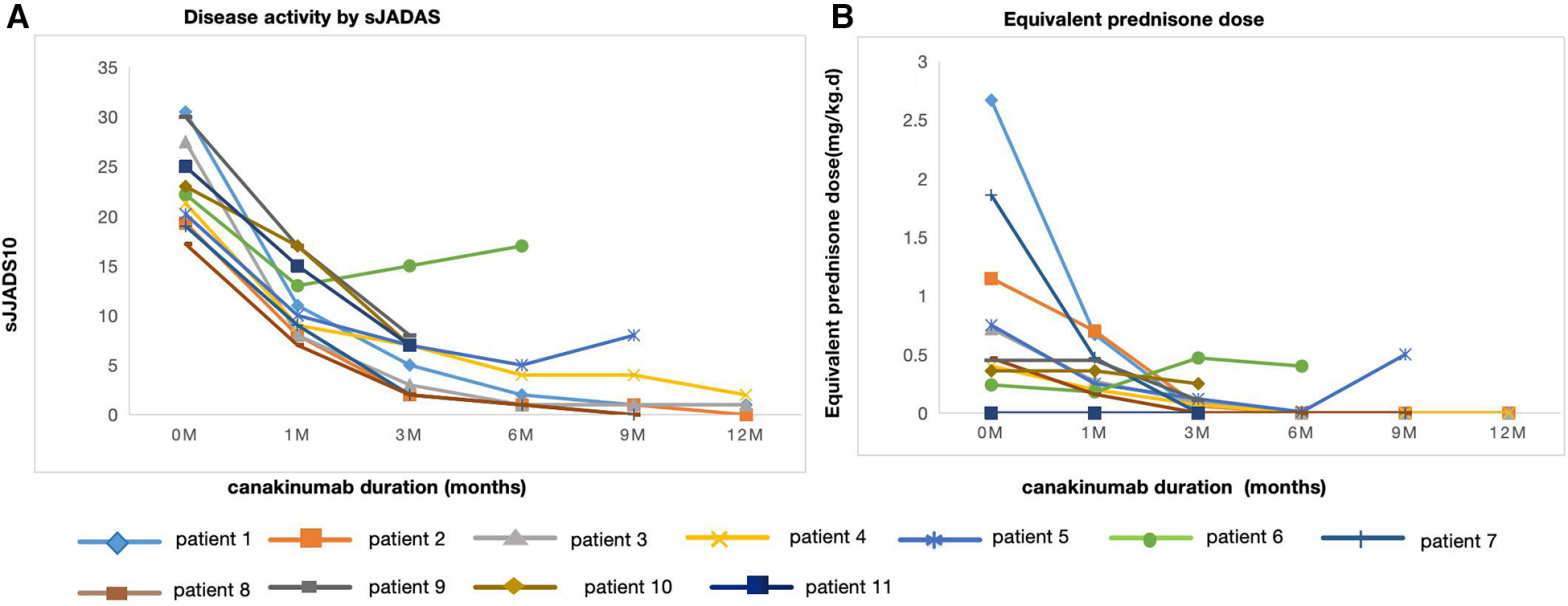
Disease activity by sJADAS and equivalent prednisone dose over time at canakinumab duration. (**A**) Disease activity assesed by sJADAS in 11 patients before (0 m) and after canakinumab at 1,3,6,9 and 12 months. (**B**) Equivalent prednisone dose in 11 patients before (0 m) and after canakinumab at 1,3,6,9 and 12 months.

**Figure 2:**
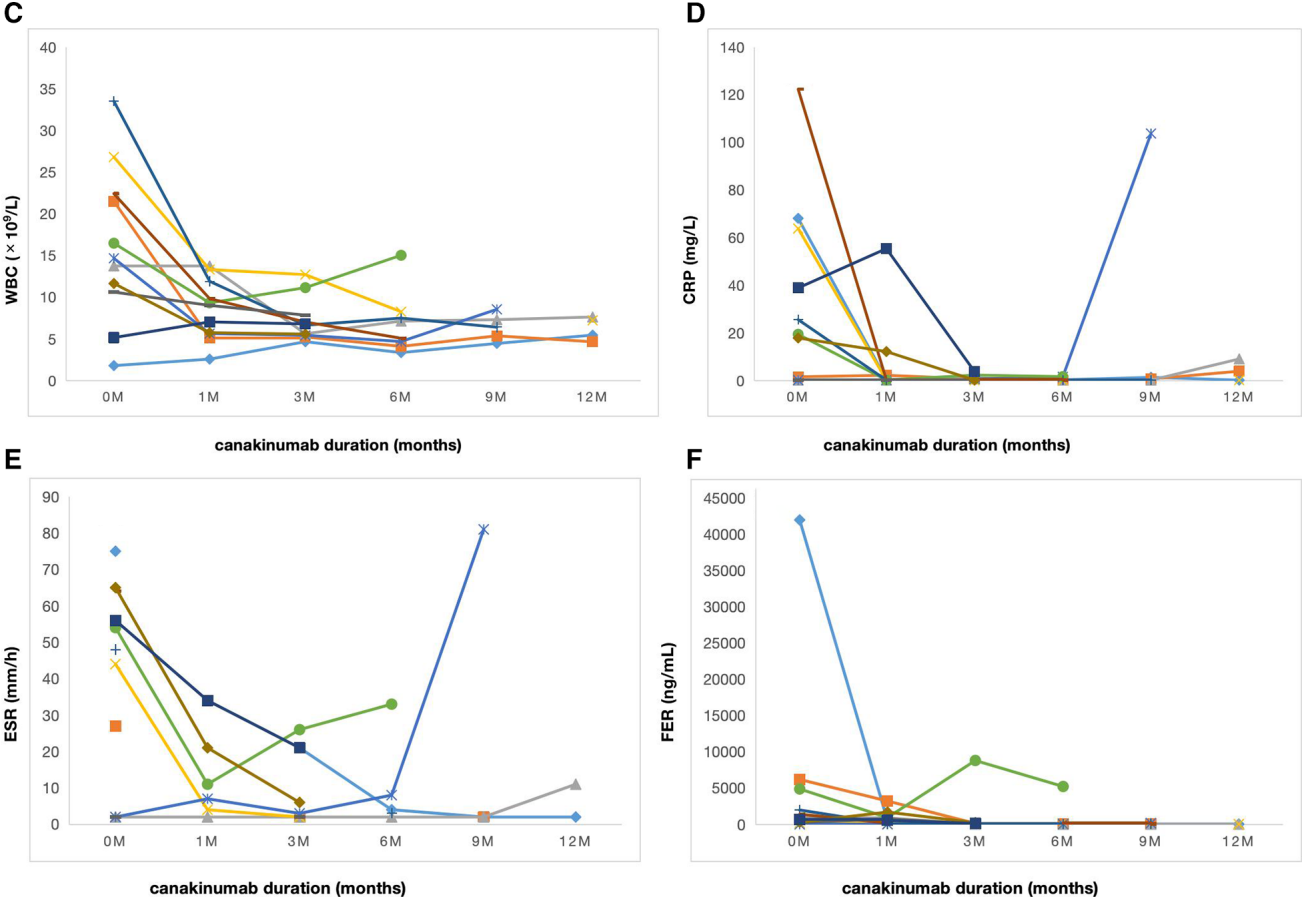
Laboratory characteristics of sJIA over time during canakinumab therapy. (**C**) WBC counts in 11 patients before (0 m) and after canakinumab at 1,3,6,9 and 12 months. (**D**) The level of CRP in 11 patients before (0 m) and after canakinumab at 1,3,6,9 and 12 months. WBC, white blood cell; CRP, C-reactive protein. (**E**) The level of ESR in 11 patients before (0 m) and after canakinumab at 1,3,6,9 and 12 months. (**F**) The level of FER in 11 patients before (0 m) and after canakinumab at 1,3,6,9 and 12 months. ESR, erythrocyte sedimentation rate; FER, ferritin.

The median duration of canakinumab treatment in this group was 9 (3–18) months. Most patients showed variable response to canakinumab treatment. After three months of canakinumab treatment, the median score of sJADAS was decreased to 7 (2, 15) (Figure 1). The median level of white blood cells (WBC) counts reduced from 14.67 × 10^9^/L to 7.5 × 10^9^/L (*p *= 0.01) (Figure 2C). The level of erythrocyte sedimentation rate (ESR) and C-reactive protein (CRP) levels decreased in majority of patients except patient 5, who experienced disease flare with increased level of CRP and ESR levels during canakinumab therapy (Figures 2D,E). Except patient 6 with persistent hyperferritinemia, majority of patients experienced rapid reduction in the level of ferritin (FER) after canakinumab therapy (Figure 2F).

Five patients (45.5%) showed complete response after six months of canakinumab treatment, all maintaining complete remission after prolonged the dose interval of canakinumab. Patient 2 and 3 used canakinumab as monotherapy without a cDMARD. Patient 1,7 and 8 used canakinumab together with MTX, all with no disease flare (Table 2).

Five patients (45.5%) showed partial response. Patient 4 showed significant improvements in arthritis and laboratory parameters, but still had occasional rash. JAKi was used concomitantly with canakinumab in Patient 9,10 and 11. Patient 9 and 10 suffered from intermittent rash. The level of ESR in Patient 11 was persistently abnormal but experienced a decrease during canakinumab therapy. Patient 5 experienced disease flare manifesting with fever, rash and arthritis with elevated level of ESR and CRP when canakinumab treatment was prolonged to once every six weeks. Although the interval dose of canakinumab was adjusted to once every four weeks and the equivalent dose of prednisone was increased, he still presented with persistent arthritis. Then he discontinued canakinumab treatment and switched to tocilizumab with MTX at the last follow-up (Table 2).

Patient 6 showed no response to canakinumab treatment. Due to persistent hyperproteinemia, recurrent disease flare, and poor compliance, he stopped canakinumab treatment after receiving seven doses. At the last follow-up, He received prednisone, cyclosporin and tofacitinib to control disease activity, but still experienced hyperferritinemia, rash, and intermittent fever episodes (Table 2).

### Corticosteroid-tapering effect

The corticosteroid-tapering effect was observed in nine patients. Six of these discontinued corticosteroids treatment after the introduction of canakinumab (Table 2 and Figure 1), including five maintaining a complete remission. Patient 11 achieved corticosteroids free before canakinumab treatment. Although Patient 5 failed to taper corticosteroids continuously due to the disease flare, he received a much lower dose of corticosteroids after canakinumab treatment. The corticosteroid-tapering effect was not observed in Patient 6 even after the introduction of canakinumab, he still presented with recurrent disease flare. Therefore, he discontinued canakinumab treatment after seven doses of this agent and switched to an initial dose of corticosteroids combined with JAKi and cyclosporin.

### Safety of canakinumab in sJIA

Canakinumab was tolerable in most patients. No severe adverse effects were observed. The most common adverse event was upper respiratory tract infection (45.6%, 5/11). Patient 1 had acute cervical lymphadenitis and Patient 5 experienced gastroenteritis. No patients discontinued canakinumab treatment due to adverse effects. Severe adverse events, notably serious infection were not observed (Table 2).

## Discussion

Herein, we firstly described the efficacy and safety of canakinumab in Chinese patients with sJIA. Most patients in the study had minimal response to tocilizumab treatment and received high doses of corticosteroids before canakinumab initiation. Most of them showed partial or complete response and had substantial corticosteroids dose reduction or discontinuation after the introduction of canakinumab. Only one patient showed no response. Ruperto et al. had described that 20% of patients achieved clinical remission at 6 months and 32% at 2 years, and the efficacy maintained up to 5 years (11). Nishimura K et al. had described that corticosteroid was discontinued or successfuly tapered in 66.7% of patients (5). Consistent with our results, these results demonstrate that canakinumab treatment may be effective and help to reduce the dose of corticosteroids in sJIA patients.

The efficacy of tocilzumab as first-line biologic therapy for children with sJIA has been practically assured. However, similar to a number of reported sJIA patients, most patients in our study discontinued tocilizumab treatment due to lack of efficacy or side effects. Patient 10 discontinued the treatment due to possible allergic reaction (abdominal pain and rash). There were no trials directly comparing the efficacy between tocilizumab and canakinumab. The latest 2021 ACR Guideline didn't recommend a priority order, taking both tocilizumab and IL-1 inhibitors as initial therapy in the treatment of sJIA (3). However, Horneff G et al. found that tocilizumab was more frequently discontinued due to intolerance compared to IL-1 inhibitors (12). Alexeeva E et al. had described that 82% of patients with sJIA achieved remission with one-year canakinumab therapy after tocilizumab treatment failure (13). Besides, He T et al. had described tocilizumab-induced hypofibrinogenemia in patients with sJIA, which was not observed in patients with canakinumab therapy (14). Therefore, canakinumab might be more effective and tolerable in patients with sJIA, especially those who do not respond to or cannot tolerate tocilizumab.

In Europe and the United States, canakinumab has been approved to treat patients aged ≥2 years with active sJIA and the recommended dose was 4 mg/(kg.d) (max 300 mg/d) every 4 weeks, while the dosage in active sJIA-related MAS was not mentioned. The median dosage of canakinumab used for the first time in our study was 4.5 (2, 8) mg/kg and the median dosage for maintenance was 4 (2, 6) mg/kg. Patient 1 received a dose of 8 mg/kg for the first time due to uncontrolled MAS, showing minimal response to the treatments in methylprednisolone pulse, etoposide, and doxorubicin liposome. Kostik MM et al. described that 50% of patients received one scheduled injection of an increased dose (2–3 times standard doses) of canakinumab with rapid resolution of sJIA-associated MAS allowing for tapering back down to standard canakinumab dosing of 4 mg/kg every 4 weeks (15). In addition, 45.5% of patients in our study were given to JAKi at the initiation of canakinumab treatment. He T et al. had reported that ruxolitinib combined with canakinumab may be useful to treat MAS and control underlying sJIA disease (10). These findings suggest that temporally up-titrated doses of canakinumab at initiation or combined with JAKi might be a potential therapy to achieve rapid resolution of sJIA-related MAS.

45.5% (5/11) of patients maintained complete response until last follow-up time, and two even received canakinumab monotherapy. 54.5% (6/11) of patients with clinical remission successfully prolonged the dose intervals of canakinumab. These findings were consistent with a randomized study, in which 67.8% (46/68) of patients achieved clinical remission through canakinumab monotherapy and majority of patients maintained clinical remission during canakinumab tapering (16). As previously reported studies have shown, infections, such as respiratory infections, were the common adverse effects, especially in young patients (17). No patients discontinued canakinumab treatment due to adverse effects. Although more attention should be paid to the complications of infection in young patients, the long-time use of canakinumab may be well tolerated in most patients.

This research had limitations on that it was a retrospective study in a single center and the sample size was small, which increased the risk of making type 2 errors in significance testing. We cannot directly attribute the efficacy of therapy solely to the effect of canakinumab, since most patients received at least one or more immunosuppressant agents. Longer follow-up and future prospective clinical trials are required to further evaluate the long-term efficacy and safety of canakinumab in Chinese patients with sJIA.

## Conclusions

Canakinumab may be an effective and safe therapeutic agent for Chinese sJIA patients, helping to reduce the dose of corticosteroids. For young children, attention should be paid to infection complications during the treatment with canakinumab. These data provide the basis for randomized control trials to evaluate the efficacy of canakinumab in Chinese sJIA patients.

## Data Availability

The raw data supporting the conclusions of this article will be made available by the authors, without undue reservation.
